# Reflecting on Behavioral Spillover in Context: How Do Behavioral Motivations and Awareness Catalyze Other Environmentally Responsible Actions in Brazil, China, and Denmark?

**DOI:** 10.3389/fpsyg.2019.00788

**Published:** 2019-06-04

**Authors:** Nick Nash, Lorraine Whitmarsh, Stuart Capstick, John Thøgersen, Valdiney Gouveia, Rafaella de Carvalho Rodrigues Araújo, Marie K. Harder, Xiao Wang, Yuebai Liu

**Affiliations:** ^1^Tyndall Centre for Climate Change Research, School of Psychology, Cardiff University Cardiff, United Kingdom; ^2^Department of Management, Aarhus University Aarhus, Denmark; ^3^Department of Psychology, Federal University of Paraíba João Pessoa, Brazil; ^4^School of English and Media Studies, Massey University Wellington, New Zealand; ^5^Fudan Tyndall Center, Department of Environmental Science and Engineering, Fudan University Shanghai, China; ^6^Independent Ethnographic Consultant London, United Kingdom

**Keywords:** behavioral spillover, pro-environmental behaviour, cross-cultural, Brazil, China, Denmark, qualitative

## Abstract

Responding to serious environmental problems, requires urgent and fundamental shifts in our day-to-day lifestyles. This paper employs a qualitative, cross-cultural approach to explore people’s subjective self-reflections on their experiences of pro-environmental behavioral spillover in three countries; Brazil, China, and Denmark. Behavioral spillover is an appealing yet elusive phenomenon, but offers a potential way of encouraging wider, voluntary lifestyle shifts beyond the scope of single behavior change interventions. Behavioral spillover theory proposes that engaging in one pro-environmental action can catalyze the performance of others. To date, evidence for the phenomenon has been mixed, and the causal processes governing relationships between behaviors appear complex, inconsistent and only partly understood. This paper addresses a gap in the literature by investigating accounts of behavioral spillover in three diverse cultural settings using qualitative semi-structured interviews. The analysis shows that while around half of participants overall who were questioned recalled spillover effects, the other half had not consciously experienced spillover. There were few significant differences across cultures, though some forms of spillover effects were reported more in some cultures than others. More environmentally engaged participants across all three countries were significantly more likely to experience spillover than those who were less engaged. Accounts of within-domain spillovers were most commonly reported, mainly comprising waste, resource conservation and consumption-related actions. Accounts of between-domain spillover were very rare. Recollection of contextual and interpersonal spillover effects also emerged from the interviews. Our findings suggest that more conscious behavioral spillover pathways may be limited to those with pre-existing environmental values. Behavioral spillover may comprise multiple pathways incorporating conscious and unconscious processes. We conclude that targeting behavioral catalysts that generate more socially diffuse spillover effects could offer more potential than conventional spillover involving a single individual.

## Introduction

Pro-environmental behavioral spillover has received renewed interest in the social sciences in recent years as a potential way of initiating voluntary environmentally responsible lifestyle change beyond that of piecemeal behavioral interventions. Behavioral spillover has an intuitive logic and appeal, yet the academic research has been limited (Thøgersen and Crompton, 2009). The majority of research comes from quantitative experiments and field studies; where spillover effects have been observed they are typically conditional (Thøgersen, 1999) with modest effect sizes (Thomas et al., 2016). Nonetheless, they may still be important, especially if they persist over an extended time period (Juhl et al., 2017), promote important behaviors (Lauren et al., 2016) or generate attitude change, such as increased acceptance of environmental policy (Thøgersen and Noblet, 2012).

A substantial volume of research has investigated behavioral spillover from the perspective of behavioral outcomes following an intervention, yet very little attention has been given to individual perceptions in the context of everyday lifestyles. There may be multiple pathways to generating observable spillover effects. While some of these processes may occur more or less unconsciously, for example, through identity change (Lauren et al., 2018), very little work has examined individuals’ conscious perspectives on the spillover phenomenon in the context of their pro-environmental behavioral motivations. Moreover, few studies have investigated behavioral spillover from a cross-cultural perspective. In this paper, we look at individual accounts of behavioral spillover in three culturally diverse nations (Brazil, China, and Denmark). In Brazil and China, factors such as rapid economic development and population growth predict a significant rise in carbon emissions in the near future (Hallding et al., 2013), while, in contrast, Denmark has made some progress in preventing further damage to its natural ecosystems and has set out a strategy to become fossil-fuel independent by 2050 (Wu, 2015). This article is one of the first to explore citizens’ experiences of spillover from a detailed, qualitative perspective. We include reflections from both environmentally engaged and less engaged citizens and evaluate the potential for spillover as a means of catalyzing wider sustainable lifestyle shifts.

Within psychology, most studies of pro-environmental behavior change apply a reasoned action model of individual behavior based on the broad assumption that individuals negotiate behavioral decision-making in rational ways. For example, the Theory of Planned Behavior (TPB) (Ajzen, 1991) asserts that behavior is driven by beliefs about the likely consequences of an action, perceived social norms, and perceived behavioral control over a given situation. Likewise, Stern’s (2000) Value Belief Norm (VBN) theory states that when behavior is not strongly constrained by contextual factors, personal norms (internalized rules or obligations to act in a certain way), become activated when valued objects (including the environment), are threatened. With reference to the wider social context in which behaviors occur, Cialdini has pioneered research on the importance of social norms in pro-environmental behavior change (for example, Cialdini et al., 1990; Cialdini and Goldstein, 2004). More recently, Community-Based Social Marketing (CBSM) has also been applied to pro-environmental behavior (McKenzie-Mohr et al., 2011). CBSM goes beyond changing individual cognitions by removing the barriers to pro-environmental actions and enhancing the benefits of engaging in order to make acting in an environmentally responsible way the rational choice. Conversely, behavioral spillover research draws mainly on “non-reasoned” theories, especially consistency theories such as Festinger’s (1957) Theory of Cognitive Dissonance and Bem’s (1972) self-perception theory. Consistency theories assume that behavior change is the outcome of people’s post-rationalization of behavior, triggered by feelings of discomfort (Thøgersen, 2004) or the increased salience of a pro-environmental self-identity (Scott, 1977; Lanzini and Thøgersen, 2014).

Behavioral spillover research is concerned with the possibility of voluntary, wider lifestyle shifts beyond piecemeal behavior change. Research on spillover builds on the idea that engaging in a behavior can, under certain circumstances, affect engagement in other actions aligned with the same goal. Spillover effects have been observed in several disciplines, including psychology, economics, sociology, and health studies from the gray literature (Austin et al., 2011). Evidence for behavioral spillover effects has emerged from research into moral self-regulation (Sachdeva et al., 2009), safety (Ludwig and Geller, 2000), and health (Devine et al., 2003), in addition to pro-environmental behavior (Lauren et al., 2018). The literature on pro-environmental spillover includes studies of *positive* and *negative* spillover effects, with a number of reviews drawing on both literatures having been published (Truelove et al., 2014; Dolan and Galizzi, 2015; Nash et al., 2017; Nilsson et al., 2017), as well as a notable review in the gray literature (Austin et al., 2011).

*Positive* behavioral spillover concerns the idea that engaging in one environmentally responsible action (and therefore an intervention targeting a specific behavior), can catalyze engagement in other behaviors (untargeted by the intervention) (Truelove et al., 2014). Engaging in one pro-environmental behavior can lead to the adoption of others (Lanzini and Thøgersen, 2014; Juhl et al., 2017; Lauren et al., 2018), including behavioral catalysts that increase engagement in more committed behaviors (Lauren et al., 2016) and increased support for environmental policy (Thøgersen and Noblet, 2012; Lacasse, 2017).

*Negative* behavioral spillover asserts that an intervention targeting one pro-environmental behavior can limit engagement in other, untargeted actions (Thøgersen, 1999; Nilsson et al., 2017). Negative relationships between pro-environmental behaviors are further suggested by studies into allied phenomena such as moral licensing (Blanken et al., 2015), and economic rebound effects (Chitnis et al., 2013). While acknowledging the complexity and ambivalence inherent in behavioral relationships, for the remainder of the paper we focus on *positive* behavioral spillover (henceforth, behavioral spillover). This is because the plurality of approaches, constructs and pathways, both between, and, indeed, within the literatures on positive and negative spillover effects, cannot be covered in sufficient depth in a single study.

There is some evidence cross-nationally to support the theory that the chance of adopting a novel pro-environmental behavior increases when behaviors are conceptually related in a Danish study (Thøgersen and Ölander, 2003; Thøgersen, 2004), share similar routines or resources in an Australian context (Margetts and Kashima, 2017) and the United Kingdom (Littleford et al., 2014). Uptake of a new behavior may also be facilitated if an individual has previously engaged in a more difficult action (Xu et al., 2018), comparable to the “*Foot-In-The-Door*” effect, in which compliance with a task performance request increases following compliance with a more difficult initial request (Scott, 1977; Truelove et al., 2014). While such findings are encouraging, they also imply that spillover effects may be limited. Other studies have observed broader behavioral shifts across different behavioral clusters, such as driving fuel efficiently and intention to reduce meat consumption in the Netherlands (Van der Werff et al., 2014), and green purchasing and increases in multiple actions including use of public transport, recycling, water and energy conservation, and volunteering for a green cause in Denmark (Lanzini and Thøgersen, 2014).

Despite such support, some of the evidence for behavioral spillover comes from self-reported intentions rather than observed behavior change (Xu et al., 2018), and from correlational study designs that cannot rule out reverse causality or the influence of common factors (Thøgersen, 2012). Longitudinal studies offer more reliable support. Thøgersen and Ölander (2003) reported on a Danish study that found associations between increased engagement in recycling and subsequent increases in organic food purchasing and public transport use measured at three time points. More recently, in a Chinese study, Xu et al. (2018) observed that engagement in household waste separation catalyzed a subsequent reduction in domestic energy consumption over a three-year period, mediated by changes in self-perception. In another study extracting purchasing behavior from supermarket scanner data covering 8000 Danish households over 20 months, Juhl et al. (2017) found that consumers who started to buy organic items in one product category subsequently purchased organic items in more and more categories over time. In addition to the adoption of new behaviors or changes in the frequency of existing environmentally responsible practices, spillover effects may occur whereby pro-environmental behavior is transferred from one context to another, such as from work to home (Rashid and Mohammad, 2011; Nilsson et al., 2017), or, in the gray literature, from one individual to another in different contexts (Austin et al., 2011). From the literature review so far, it appears that while some evidence comes from laboratory studies, behavioral spillover can also occur in natural settings comprising a variety of behavioral catalysts and effects; but it is not a consistent phenomenon, is difficult to detect and it does not appear to operate in a uniform way.

As well as documenting behavioral outcomes following an intervention, research has sought to understand the processes underpinning observed catalytic relationships. Prospective pathways to spillover include desire for behavioral consistency (Thøgersen, 2004), change in self-identity (Lauren et al., 2018), increased knowledge and self-efficacy (Thøgersen, 2012), heightened environmental concern (Carrico et al., 2018), and strength of felt responsibility to act (Lacasse, 2017).

Identity-based approaches have gained traction and are based on the idea that people infer how to act in a given situation through perceived self-identity and past behavior (Bem, 1972). Engaging in pro-environmental behavior can generate a ‘greener’ sense of self, which increases the likelihood of acting in ways consistent with this identity in future (Lauren et al., 2018). Increasing green self-perceptions can increase intentions to act environmentally responsibly, as found in a Dutch study (Van der Werff et al., 2014; see also Cornelissen et al., 2008) as well as increase environmental concern and boost support for environmental policy as found in a US study (Lacasse, 2016). Following the introduction of a single-use plastic bag charge in Wales, people’s environmental self-perceptions were stronger than before the charge (Poortinga et al., 2013). In the United States, Carrico et al. (2018) failed to detect a change in green self-perception following pro-environmental behavior change. They suggest that the way in which green identity is manipulated may be critical in whether spillover is produced.

Unsurprisingly, engaging in pro-environmental behavior can increase relevant knowledge, skills and experience in ways that facilitate the adoption of other behaviors, as found in Denmark and the United Kingdom (Hutton, 1982; Thøgersen, 1999). Familiarity with eco product labels also predicted subsequent increased purchasing of ecological products in a Danish supermarket study (Thøgersen et al., 2010). Enhancing citizens’ pro-environmental literacy and skills can therefore increase the potential for wider pro-environmental engagement (Thøgersen, 2012). Related to knowledge and experience, self-efficacy (a subjective perception of one’s capacity to act in a given situation; Bandura, 1977), offers another pathway to behavioral spillover. An intervention designed to promote energy conservation by a German energy provider was associated with a range of behavioral spillovers (including reducing meat consumption, reducing car use, and donating to an environmental cause), in which spillover was mediated by change in self-efficacy (Steinhorst et al., 2015). Self-efficacy has also been observed to mediate behavioral spillover from less committed to more committed water conservation actions in Australia (Lauren et al., 2016). However, in a subsequent study looking at different behavioral relationships (Lauren et al., 2018), it was green self-identity rather than self-efficacy that mediated spillover between green household actions.

Spillover effects may be more consistently measured when individuals hold pre-existing pro-environmental values. Priming pro-environmental values increases the likelihood of engagement in environmentally responsible behavior (Schultz and Zelezny, 1998) and increases the strength of spillover relationships (Thøgersen and Ölander, 2003). Thøgersen and Crompton (2009) note that prioritizing or valuing the environment may be a necessary prerequisite for behavioral spillover, therefore spillover may be limited to more environmentally engaged citizens. The phenomenon is rendered even more complex by variation in individual behavioral engagement in different contexts. For example, pro-environmental commitments may be relaxed when on vacation (Barr et al., 2010), or when roles and responsibilities between one context and another are perceived to differ (Maki et al., 2016).

Little research has utilized qualitative approaches in studying spillover. Schütte and Gregory-Smith (2015) and Barr et al. (2010) interviewed German and British holidaymakers, respectively, concluding there was little evidence for spillover of domestic pro-environmental actions between home and holiday contexts. In the gray literature, Austin et al. (2011) conducted 20 interviews with behavior change practitioners in the United Kingdom and provide anecdotal evidence that engagement in green behaviours catalyzes other actions. Wonneck and Hobson (2017) also used interviews, concluding that participation in a municipal food-waste recycling program in Canada increased engagement in recycling and environmentally responsible food shopping practices. Finally, Dumitru et al. (2016) analyzed interviews, focus groups and evidence from text documents in Italy and Spain, reporting contextual spillover of pro-environmental values from the workplace (a green energy company) to its employees.

We are unaware of any papers taking a qualitative, cross-cultural approach to behavioral spillover and this paper addresses a significant gap in the literature. Our approach situates accounts of behavioral spillover in the wider sociocultural context, to linked factors beyond the ecological (Howell, 2013). CBSM theory highlights the importance of wider psychological and structural barriers constraining the adoption of pro-environmental behavior, therefore attending to perceived barriers to spillover might offer windows of opportunity for intervention (McKenzie-Mohr et al., 2011). We investigate whether citizens are conscious of behavioral spillover effects as significant motivators of their environmentally responsible practices. Culture exerts a powerful effect on pro-environmental behavior (Adger et al., 2013), shaping people’s value emphasis (Leonard et al., 2013; Schwartz, 2014), and the patterns and routines of everyday life (Sztompka, 2008; Gram-Hanssen, 2011).

We evaluate the potential for behavioral spillover as a pathway to more environmentally sustainable societies, pointing out that understanding behavioral spillover in culturally diverse settings is crucial for designing effective interventions to bring about wider lifestyle shifts, especially in countries where environmental policy and infrastructure are less developed and where behavioral catalysts could be better tailored to optimize urgently needed lifestyle change. Encouraging even modest lifestyle shifts could significantly reduce a nation’s environmental impacts (Dietz et al., 2009). While behavioral spillover effects have been observed in Europe (Thøgersen and Ölander, 2003), the United States (Truelove et al., 2016), Asia (Rashid and Mohammad, 2011) and Australia (Lauren et al., 2016), Spillover might be more common in nations where external factors such as cultural values, education, environmental infrastructure, and environmental services are more supportive of sustainable lifestyle choices, as found in a piece of research comparing differences between Mexico, United States, Spain and Brazil (Vicente-Molina et al., 2013). As self-identity appears germane to spillover processes, cultural differences in self-construal (English and Chen, 2007) may affect the transfer of pro-environmental behavior through identity channels. While individual personal values may vary within a given setting, cultural values, such as those linked to identity, express the integration of ideas, norms, beliefs and values within a society that contribute to individual perspectives and underpin behavior (Oreg and Katz-Gerro, 2006). A qualitative cross-cultural approach to behavioral spillover can also serve to identify gaps between scientific definitions of behavioral spillover and the more experiential perspectives of citizens (Lowe et al., 2006), in line with the active, functional ways that individuals construct their worlds (Potter, 1996), and in which theoretical delineations and boundaries are blurred and do not necessarily match conventional behavioral schematics (Rudiak-Gould, 2012).

Following our review of the literature, 5 research questions are set out as follows:

1. Do citizens in diverse cultural contexts recollect personal experience of positive behavioral spillover?
2. If so, do recollections of behavioral spillover differ between these cultures?
3. Does degree of environmental engagement influence experience of positive behavioral spillover?
4. What kinds of behavioral spillover effects emerge in citizens’ accounts and which behaviors are involved?
5. Are there any reported barriers to spillover?

## Materials and Methods

This section details the design and procedure used in the study, which was approved by the Cardiff University’s School of Psychology Research Ethics Committee. The design was based on a set of 96 semi-structured qualitative interviews with more and less environmentally engaged citizens in each of the three countries. Interviews were designed to elicit perceptions of green lifestyles and behavior, including recollections of behavioral spillover as a reason for engaging in pro-environmental actions.

### Participants

Interviews were conducted between March 2015 and April 2016. A purposive sampling strategy (Silverman, 2015) was utilized to ensure that each country sample included a range of environmental values and socio-demographic characteristics (including gender and age). All participants were aged 18+ and comprised two distinct groups. To generate a range of environmental perspectives we recruited in two ways; first of all, we approached potential academic collaborators to help recruit citizens whose environmental values were broadly reflective of the “average” citizen. To do so, we advertised the study as a “*behavior and lifestyle perceptions*” study and avoided explicitly mentioning “*the environment*.” In addition, we also approached environmental organizations to recruit another subsample of citizens who were more environmentally engaged.

In Brazil, fieldwork took place in the capital Brasília (population 2.481 million), and João Pessoa, on the North East coast in the State of Paraíba (population 720,000), during March/April 2015. In total, 35 citizens participated. The less environmentally engaged group comprised residents of João Pessoa, who were recruited by collaborators at the Federal University of Paraíba. The study was advertised locally asking interested residents to get in touch. Participants were subject to a brief screening procedure to ensure they were 18+ and did not work in the environmental sector or have any heightened pro-environmental commitments or values, and to ensure we had some variation in terms of factors such as gender^1^ and age (*n* = 17). The environmentally engaged group were recruited by collaborators at the offices of the World Wildlife Fund for Nature (WWF) office in Brasília. An advert for the study explicitly mentioning an interest in employees who were environmentally engaged was circulated internally (*n* = 18). This group were also screened to ensure that participants were environmentally committed in their lifestyles (as some employees worked for WWF in a more technical capacity and might lack such commitment), as well as to ensure some variation in terms of gender and age. See Table 1 for participant demographics.

**Table 1:** Participant demographics for all subsamples.

		Less environmentally engaged		More environmentally engaged		Subsamples combined	
**Brazil**							
*Gender*	*Female*	10	58.8%	9	50%	19	54.3%
	*Male*	7	41.2%	9	50%	16	45.7%
*Age group*	*18–24*	4	23.5%	0	0%	4	11.4%
	*25–34*	3	17.6%	5	27.8%	8	22.9%
	*35–44*	2	11.8%	10	55.6%	12	34.3%
	*45–54*	4	23.5%	3	16.7%	7	20%
	*55–64*	0	0%	0	0%	0	0%
	*65+*	4	23.5%	0	0%	4	11.4%
**Denmark**							
*Gender*	*Female*	9	64.3%	12	70.6%	10	32.3%
	*Male*	5	35.7%	5	29.4%	21	67.7%
*Age group*	*18–24*	4	28.6%	4	23.5%	8	25.8%
	*25–34*	7	50%	8	47.1%	15	48.4%
	*35–44*	2	14.3%	0	0%	2	6.5%
	*45–54*	1	7.1%	3	17.6%	4	12.9%
	*55–64*	0	0%	0	0%	0	0%
	*65+*	0	0%	2	11.8%	2	6.5%
**China**							
*Gender*	*Female*	9	60%	7	46.7%	16	53.3%
	*Male*	6	40%	8	53.3%	14	46.7%
*Age group*	*18–24*	0	0%	3	20%	3	10%
	*25–34*	12	80%	7	46.7%	19	63.3%
	*35–44*	1	6.7%	5	33.3%	6	20%
	*45–54*	1	6.7%	0	0%	1	3.3%
	*55–64*	0	0%	0	0%	0	0%
	*65+*	1	6.7%	0	0%	1	3.3%


The city of Aarhus on the East coast of the Jutland Peninsula (population 336,000), was the setting for the Danish fieldwork in August/September 2015 (*n* = 31). The less environmentally engaged group were recruited by collaborators at Aarhus University who advertised the study online (*n* = 14). After initially approaching WWF Denmark (who were unable to collaborate), collaborators at Aarhus University also recruited the more environmentally engaged group by posting an advert on the Aarhus Sustainable Initiatives Network (*n* = 17) Participants constituted volunteers, employees and freelance consultants working locally in the environmental sector (see Table 1).

In China, interviews were conducted in and around Shanghai (population 24.18 million), during March 2016 (*n* = 30). The less environmentally engaged group were recruited through an online advert, by an ethnographic research collaborator who was familiar with the city and collaborators at Fudan University (*n* = 15). The environmentally engaged group were recruited by the ethnographic collaborator who advertised the study on the “Shanghai Green Initiatives” network on the “WeChat” social media app (*n* = 15). Participants comprised volunteers, employees and freelance environmental consultants working locally in the environmental sector (see Table 1).

### Procedure

Following recruitment, individuals were invited to participate in an interview to discuss aspects of their day-to-day behaviors and lifestyle. As a rule, interviews in all countries were held at the collaborating academic institution or organization; however, for some participants who were unable to make the journey but wanted to participate, the interview team agreed to hold interviews elsewhere, including cafes, workplaces or participants’ homes, whichever was most convenient.

In Brazil, all interviews with the less engaged group were held in a private interview room at the University of Paraíba in João Pessoa. All interviews with more engaged participants took place in a private meeting room at WWF in Brasília. In China, 13 of the interviews with less engaged participants were held in a rented meeting room in the center of Shanghai and the remaining 2 took place elsewhere (one in a café and one in the participant’s home). For the more engaged group, 9 interviews took place in the rented meeting room or in a meeting room at Fudan University, while 6 were held in participants’ workplaces. In Denmark, all interviews with the less engaged group took place at Aarhus University. For the more engaged group. 7 interviews were held at the university, while the other 10 interviews took place in participants’ workplaces.

#### Ethical Considerations for Working Across Three Countries

While interviews are commonly used in social research, the methodology carries its own important procedural and ethical implications. Inequitable power relations are unavoidable in academic research where the interaction is primarily directed by the researcher (King et al., 2018). Interactional identities are compounded by factors such as gender, ethnicity, socioeconomic status and education, which may be overt or covert (Anyan, 2013). Cultural assumptions imposed through interview protocols, questioning and instrumentation can potentially cause offense and discomfort to participants situated in other cultures; such inequitable dynamics can also diminish the value of the information obtained (Brinkmann and Kvale, 2008). It is critical that cross-cultural research teams consider ethical issues not only in terms of the interview interaction itself, but to procedural issues prior to the interview interaction (including protocol design, question wording and recruitment), and ongoing reflections following the interview (including analysis, reporting findings and dissemination of research) (Hoover et al., 2018).

In designing the interview protocol, we worked closely with in-country collaborators to ensure not only that the protocol and question wording were designed to elicit the topics in which we were interested, but to address issues of culturally imposed bias (such as making assumptions about environmental conditions, values and lifestyle practices of those within a given culture). All interview materials were double-translated into the local language(s). For balance and to reduce potential cultural and gender imbalance that might otherwise constrain trust and disclosure, particularly for female participants (Campbell and Wasco, 2000; Sikes, 2018), the interview team comprised the same male researcher (lead author) and a different female translator in each country. The female translator played an active part in the interaction as opposed to simply translating questions and responses, introducing additional questions, checking understanding and elaborating on culturally relevant issues for clarification. Having a cultural “insider” as part of the team helped facilitate trust and disclosure, while the presence of a cultural “outsider” generated greater insight into the participant’s world by rendering the familiar strange (Dwyer and Buckle, 2009). The presence of a researcher from another culture also occasionally led to a richer exploration of perspectives linked to economic globalization, resource inequities and sources of environmental harms beyond geographical borders. While a translator was present, some participants expressed a willingness to conduct interviews in English or switched between English and their native language (for example, if they were unable to explain a point in English). We acknowledge that translation imposes an additional level of interpretation on an utterance (Caretta, 2015), therefore we have tried insofar as possible, to analyze accounts based on participants’ direct speech rather than the translator’s interpretation.

As mentioned, for practical reasons it was not always possible to interview participants at the collaborating institutions. In such cases we took the pragmatic decision to stage interviews in other locations, such as workplaces and homes. In doing so, we acknowledge that space and place are active and influential factors in negotiation interactions between researcher and research participant (Gagnon et al., 2015). Before conducting an interview in an alternative location, we ensured that spaces were available in which participants could discuss issues confidentially without being in the direct gaze of, overheard by, or interrupted by others. We also applied this rubric to interviews that took place in collaborating academic institutions. Allowing participants greater flexibility to choose their preferred location also served to engender a more equitable relationship with participants (Gagnon et al., 2015). We noted that when conducting interviews in participants’ homes, the home itself sometimes served as an exemplar of lifestyle discussions in which participants illustrated their accounts with reference to their home interiors, gardens and wider surroundings. We also noted that in workplace interviews, participants sometimes referred to documents and other office procedures or apparatus (such as air conditioning systems or office recycling systems), in discussions. This enriched fieldnote records and would not have been possible if held in more neutral academic institutions.

#### Analytic Approach

Written, informed consent (in the local language) was sought from all participants prior to interview. Interviews took approximately 1–1.5 h to complete, in which the interview team covered a set protocol of basic questions in all three countries for meaningful comparison, but also allowing for follow-up questions and the exploration of issues that were more culturally specific to each country. Therefore, the flexibility of the semi-structured interview method was advantageous in that it could be applied to multisited cross-cultural contexts (Hagaman and Wutich, 2017), as well as allowing the generation of more detailed, culturally specific context (McIntosh and Morse, 2015). At the end of each interview, participants were provided with a verbal and written debrief (in the local language), along with researcher contact details in case of further questions.

An episodic narrative approach was used to explore participants’ lifestyles, which seeks to ground perceptions and experiences as lived narratives within the wider society and culture (Flick, 2000; Jovchelovitch and Bauer, 2000). The episodic interview method is a form of narrative interviewing that elicits snapshot descriptions of particular episodes or features in a person’s life as a way of making sense of the world. The questions in the interview protocol sought to contextualize accounts rather than to generate more abstract responses, as this risked neglecting wider socioculturally relevant issues. The preset interview question list appears in Supplementary Appendix A. While the protocol explored a range of environmentally salient issues, this paper is primarily focused on responses elicited by the question, “*Can you remember in the past whether doing something that was good for the environment caused you to then do another environmentally-friendly behavior*?,” though we looked for examples of spillover throughout each transcript.

Interviews were audio-recorded and subsequently translated and transcribed. Written field notes were also recorded throughout the interaction. An “*in-interview*” system of translation was employed in which questions and responses were translated to and from English by the translator (except where participants preferred to speak in English). Another layer of translation was imposed at the transcription stage. In the analysis section, quotes are labeled “*Direct*” if spoken in English, or “*Transl.*” if translated from another language (either by the translator in the interview or during transcription). The interview audio and texts were analyzed using NVivo 11, supplemented by written field notes. We then used a system of template analysis to code the texts, as template analysis is particularly suited to identifying themes in both essentialist and constructionist analyses (Brooks et al., 2015).

## Results

In the following analysis, where a feature of interest is applicable across more than one country, for brevity we have illustrated this feature using a single extract and alluded to its occurrence in other cultural settings within the text. The analysis proceeds by summarizing the proportions of participant responses relating to spillover across the three countries. We then move on to categorize the different kinds of behavioral spillover emerging from elicited discussions about experience of spillover. As mentioned above, our questions focused on a range of conscious positive spillover effects and potential barriers to spillover.

### Personal Accounts of Positive Behavioral Spillover

In anticipation (based upon conclusions from prior studies) that spillover effects appear ephemeral and difficult to detect, we expected that participants would be unlikely to initiate talk of behavioral spillover themselves (particularly as at least some spillover processes are unselfconscious, therefore people may not necessarily be aware that an initial behavior led to a heightened environmental goal salience or a change in self-identity which then led to other environmentally responsible actions), the analysis is focused on responses to a single question in the interview designed to elicit recollection of spillover. It is therefore important to note that the analysis captures more subjective self-reports of spillover effects and not the less conscious processes that are also of relevance to spillover pathways.

#### Participants Recall Experiences of Behavioral Spillover

To address our first research question we aggregated and compared responses to the question of whether spillover had ever occurred, across countries. Table 2 summarizes the proportions of participants who were directly questioned about spillover (some were not asked due to time constraints) and the proportions of those recalling and not recalling spillover. As discussed in the previous section, accounts of spillover did not emerge spontaneously from the interviews. The majority of participants were directly questioned about spillover. Among those who were directly asked, exactly half recalled an experience they considered to be analogous to spillover.

**Table 2:** Frequencies of reports of spillover reported by participants in Brazil, China, and Denmark.

Sample *Less engaged (-) More engaged (+)*	*N*	Directly questioned^a^	Not questioned	Recalling spillover	Recalling spillover (% of those questioned)	Not recalling spillover	Not recalling spillover (% of those questioned)
*Brazil (-)*	17	14 (82.35%)	3 (17.65%)	4 (23.53%)	28.57%	10 (58.82%)	71.43%
*Brazil (+)*	18	13 (72.23%)	5 (27.77%)	10 (55.56%)	76.92%	3 (16.67%)	23.08%
*Brazil All*	35	27 (77.14%)	8 (22.86%)	14 (40.0%)	51.86%	13 (37.14%)	48.14%
*China (-)*	15	13 (86.67%)	2 (13.33%)	4 (26.67%)	30.77%	9 (60.0%)	69.23%
*China (+)*	15	13 (86.67%)	2 (13.33%)	11 (73.34%)	84.62%	2 (13.33%)	15.38%
*China All*	30	26 (86.67%)	4 (13.33%)	15 (50.0%)	57.69%	11 (36.67%)	42.31%
*Denmark (-)*	14	14 (100%)	0 (0%)	2 (14.29%)	14.29%	12 (85.71%)	85.71%
*Denmark (+)*	17	17 (100%)	0 (0%)	11 (64.71%)	64.71%	6 (35.29%)	35.29%
*Denmark All*	31	31 (100%)	0 (0%)	13 (41.94%)	41.94%	18 (58.06%)	58.06%
*All countries*	96	84 (87.5%)	12 (12.5%)	42 (43.75%)	50.00%	42 (43.75%)	50.00%
*All countries (-)*	46	41 (89.13%)	5 (10.87%)	10 (21.74%)	24.39%	31 (67.39%)	75.61%
*All countries (+)*	50	43 (86.0%)	7 (14.0%)	32 (64.0%)	74.42%	11 (22.0%)	25.58%


*^*a*^*Refers to whether a participant was explicitly asked “Can you remember in the past whether doing something that was good for the environment caused you to then do another environmental behavior?”**

Table 3 breaks down reports positive spillover effects into discrete categorizations based on the academic literature. Overall, the most commonly reported type of spillover effects reported were within behavioral domains (i.e., between behaviors within the same cluster). The second most commonly reported effects were those that did not fall within conventional academic definitions of spillover. The category refers to responses citing other behavioral motivations (for example, formative experiences when young, changes in personal circumstances and other experiences) as catalysts, rather than engagement in a specific behavior. A range of other spillover effects were also found but these were less commonly reported than within-domain effects. These included contextual, interpersonal and between-domain spillover effects. Finally, 4 reports of positive behavioral spillover were unclear in terms of the behaviors involved and were counted separately.

**Table 3:** Categorization of subjective spillover effects reported in the interviews.

Sample *Less engaged (-) More engaged (+)*	*N* (recalling spillover)	Positive spillover (within-domain)	Positive spillover (between-domain)	Positive spillover (behaviors unspecified)	Contextual spillover	Interpersonal spillover	Other
*Brazil (-)*	4	0	0	0	0	0	4
*Brazil (+)*	10	2	0	1	2	4	1
*Brazil All*	14	2	0	1	2	4	5
*China (-)*	4	2	0	1	1	0	0
*China (+)*	11	5	1	2	3	0	0
*China All*	15	7	1	3	4	0	0
*Denmark (-)*	2	1	0	0	0	0	1
*Denmark (+)*	12	6	1	0	1	0	4
*Denmark All*	14	7	1	0	1	0	5
*All countries*	43	16	2	4	7	4	10
*All countries (-)*	10	3	0	1	1	0	5
*All countries (+)*	33	13	2	3	6	4	5


#### Differences in Recall of Positive Behavioral Spillover Across Cultures

We found both differences and similarities in reports of spillover across cultures. Table 2 shows that in Brazil and China the majority of participants were directly questioned about spillover, while all participants in Denmark were directly questioned. Of these, over half of participants in China and Brazil recalled having experienced positive spillover, though less than half of Danish participants recollected spillover having happened to them. The largest proportion of spillover accounts came from China and the smallest came from Denmark.

Table 3 shows that despite the differences in sample sizes and the proportions of participants directly questioned about spillover, frequencies of recollections of within-domain spillover in each country were almost identical. Within-domain spillover effects were the most commonly reported categories in China and Denmark while in Brazil the most common type of account related to “other” motivations. Recollections of between-domain spillover were so infrequent that meaningful cultural comparisons cannot be drawn, other than to say that catalytic effects from one behavioral cluster to another were extremely rare in all three countries. Similarly, reports of other spillover effects were too uncommon to infer cultural differences. However, contextual spillovers were more frequently reported in China than in Brazil and Denmark, while interpersonal spillover effects were only reported in Brazil. In addition, while Chinese participants did not report other behavioral motivations as spillover effects, those in Brazil and Denmark reported the same numbers of accounts in which behavior was catalyzed by non-behaviors. More detailed discussion of the different types of spillover and further examples of cultural differences, along with quotes can be found in the section on “Personal Accounts of Different Types of Positive Spillover Effects”.

#### Behavioral Spillover Effects Are More Common Among the Environmentally Engaged

As shown in Table 2, accounts of behavioral spillover were far more common among environmentally engaged participants than those who were less engaged, regardless of cultural context. For both more and less engaged groups, the majority were directly questioned about spillover. Of those questioned, the highest proportion of accounts of spillover effects came from more engaged participants in China, followed by their counterparts in Brazil and Denmark, respectively. With reference to those less environmentally engaged, fewer accounts emerged from Danish participants than those in Brazil and China.

Between-domain spillovers were reported mainly by more environmentally engaged groups, while the rare examples of between-domain spillover came exclusively from the more engaged groups (in China and Denmark). All but one example of contextual spillover came from more engaged groups; similarly, all examples of interpersonal spillover came from the more engaged group in Brazil (Table 3). Of course, while the pathways to spillover bore some similarity across cultures, these accounts were also grounded within their specific cultural contexts. We now move on to discuss accounts of behavioral spillover effects in more detail.

### Personal Accounts of Different Types of Positive Spillover Effects

In the following sections we provide a more detailed qualitative analysis of accounts of positive behavioral spillover, illustrated with examples from the interviews.

#### Recollections of Within-Domain Spillover Effects Involving Common Domestic Actions

Reports of positive spillover in the interviews emerged from participants in all three countries studied. Where spillover effects were reported, they most commonly involved relationships between two *related* actions, or an increase in the *frequency* or *range* of a single behavior. Behaviors reported in accounts of spillover in the interviews were mainly in the private sphere and drew on a limited range of behavioral clusters. In all three countries, spillover relationships principally drew on clusters comprising *waste* (for example, littering, recycling and composting) and *resource conservation* (such as reducing energy or water use) practices practiced domestically. In Denmark, in addition to waste and resource conservation, some participants also referred to spillovers involving organic consumption and the occasional public-sphere action, such as volunteering for an environmental organization or community litter-pick (see below). These spillover effects typically involved an extension of the initial behavior, such as buying more organic products, reusing more items or picking up litter elsewhere, as opposed to catalyzing different behaviors. The following extract gives a flavor of within-domain spillover from Brazil. In the account the participant describes how consciously reflecting on existing efforts to limit paper towel use was attributed to a motivation to subsequently reduce paper waste by storing documents on the computer rather than printing them:

**Table:** 

Researcher:	*Can you remember if, say, doing one environmental behavior – could be any of them – caused you to then later do another? Do you think that ever happened?*
Participant [*Direct*]:	*Yeah. The waste of towels. For me it was important. So I started to think of each paper that I threw, each paper that I used, not to use – not to print things that – just for printing. Use more the computer storage in the computer, not printing documents. As you can see I don’t have things. Everything is in my computer.*
	(B18 more engaged group; Brasilia).

In reflecting on the shift from saving paper towels to avoiding printing on the computer, the speaker explains that the initial behavior was *personally important*. The account also suggests that the initial behavior was consciously (as opposed to habitually) performed, which is used to explain the process by which they came to adopt a new behavior with the same goal. Stating that it was possible to use an alternative form of storage (i.e., storing documents on the computer rather than as hard copies), suggests that aligning behavior consistently depends to some degree on the availability of viable alternatives in switching to more sustainable practices.

As mentioned above, in addition to the adoption of a new behavior, within-domain spillover effects not only involved situations where engaging in one action catalyzed another discrete behavior within the same cluster, but also an increase in the frequency or range of an existing behavior over time. In the next extract from Denmark, the speaker talks about organic shopping practices and a spillover effect in which organic consumption had expanded over time to include an increasing array of products:

**Table:** 

Researcher:	*Can you remember a time in the past where you did one environmental behavior and as a result of doing that it caused you to do another environmental behavior? So one behavior leading to another?*
Participant [*Direct*]:	*Maybe perhaps as I said in the beginning, that – being more aware of, for example, in the beginning buying organic eggs, for example. I think that was the first thing I was aware of, or was aware of and quite – it was important for me to buy organic eggs. Then after that it was like dairy products, milk and so on. Then I’m starting to look at other products as well. I don’t know – also that there’s a bigger – there’s a lot more products – you’re able to buy a lot more products that are organic than two or three years ago. Then I started to look at clothes … But at least the awareness of buying like environmentally responsible products had led to also buying socially responsible products. So maybe I have made a shift toward that as well, that had led to that.* (B2 more engaged group; Aarhus).

Like the previous account, the speaker constructs organic purchasing as a *conscious* and *deliberate* activity that centers on a personally salient goal. Accounting for the spillover effect relies on both awareness and the increasing availability of viable organic alternatives to conventional products.

In addition to reports of spillover effects from one behavior to another within the same cluster, examples emerged where performing a behavior catalyzed the motivation to engage with others and discuss environmental issues or encourage other people to engage in actions with the same goal. However, such examples were limited and came only from participants in Brazil and China and only from the more environmentally engaged groups in those countries. In the following example from Brazil, the speaker explains how engaging in a collection of unspecified pro-environmental actions had led them to engage more with others:

**Table:** 

Researcher:	*So I guess doing those behaviors has affected other areas of your life, as you’ve said. Has it led to you doing other things? Maybe being involved in things or other behaviors?*
Participant [*Direct*]:	*Yeah. I think I talk more about the topic with other people. Not trying to be a teacher but trying to understand how – why people don’t think on their impact. This is one point. Yeah. I think talking to other people not in a way that you are teaching them is the way that you bring people to the discussion*. (B2 more engaged group; Brasília).

Within the above account, in discussing engaging others on environmental issues, the speaker stresses that they do not wish to instruct other people, but to gain an insight into why other people are less conscious in reflecting on the environmental relevance of their behavioral decision-making. This is linked to a concern that trying to teach others will drive them away from the issue rather than draw them in. We highlight this type of example because this type of spillover effect offers significant potential as a means of generating wider engagement beyond that of the adoption of one behavior on the strength of another, for a single given individual. We now move on to discuss some rarer examples of behavioral spillover *between* different behavioral clusters.

#### Recollections of Between-Domain Spillover Effects

If behavioral spillover generates wider lifestyle shifts through spreading activation, one might expect to observe catalytic effects between environmentally responsible actions in different behavioral clusters. However, only a couple of examples of between-domain spillover were recorded in the interviews. Both came from more environmentally engaged participants in China and Denmark (see Table 3). In the first extract from Shanghai, the speaker explains how walking catalyzed the motivation to increase consumption of vegetables; though both actions were driven not by pro-environmental goals, but by goals linked to health outcomes:

**Table:** 

Researcher:	*Can you ever remember a time in the past where you did a behavior that was good for the environment, and because of doing that it led you on to do another thing that was good for the environment?*
Participant [*Transl.*]:	*…So one example he gave is when he was walking…Yeah, just walking, and he will think a lot of things, such as the health. So when he thinks about health, he eats more. vegetables to be a vegetarian. When he is healthy, then he thinks probably more exercise. He’s pursuing a comfortable life now.* (B10 more engaged group; Shanghai).

In trying to become a healthier person, engagement in an initial action aligned with a personally salient goal is constructed as generating a greater conscious awareness of other health-related actions while engaged in that behavior. This, in turn, motivated the intention to make dietary changes. In addition, toward the end the speaker explains that progress toward the desired goal (becoming healthier) increases motivations to think about doing more (exercise). The extract shows how pro-environmental behaviors can have co-benefits such as improving health. Essentially, consciously focusing on a non-environmental goal (with environmental co-benefits) may lead to between-domain spillover effects in pursuing that goal.

The other example of between-domain spillover bore a similarity to the previous example in that the manifest process governing the spillover effect was attributed to a non-environmental goal; having a simpler and less expensive lifestyle:

**Table:** 

Researcher:	*Can you remember a time where – in the past where you did one behavior that was good for the environment, and as a result of doing that behavior you did another behavior that was good for the environment?*
Interviewee [*Direct*]:	*Yeah. I cannot tell a concrete example, but it’s – I think all the things with (energy-efficient) houses and electronic cars and – I think that’s – they had influenced each other. So because of – and the goal with having the easy life without lust, but having like a house who is cheap to run, having a car who is like easy to run, and there was a guarantee and everything is just easy.* (B1 more engaged group; Aarhus).

The environmentally friendly behaviors that formed the focus of the spillover relationship (an energy-efficient home and an electric vehicle) remain undefined in terms of their causal direction (i.e., which behavior was the catalyst, and which behavior was catalyzed), though the speaker acknowledges the difficulty in recollecting a clear example in line with the expressed difficulty in recalling spillover more generally.

#### Recollections of Contextual Spillover Effects Between Work and Home

Another variant of behavioral spillover, termed contextual (Nilsson et al., 2017), or situational spillover in the gray literature (Austin et al., 2011), was reported in all three countries, albeit rarely. We found limited evidence for two kinds of contextual spillover in the interviews (where a behavior is performed by an individual in one context and then another, and where a behavior is transfered between different individuals across contexts). The few examples of contextual spillover that came up in the interviews were reported almost exclusively by more environmentally engaged participants. Two types of context came up in these accounts. One involved the transfer of behavior between work and home. Here a Danish participant explains how working in the environmental transportation sector had influenced more sustainable travel decisions outside of work:

**Table:** 

Participant [*Direct*]:	*I’m starting also to think about how you transport yourself.*
Researcher:	*Transport, yeah?*
Participant:	*Yeah. But that has something to do with my work, where we are quite involved in the whole transport sector thing, because we know how great a deal that counts for CO_2_ emissions. So in my professional – or in my job I work with how we can make intelligent transport systems to save energy and let out less CO_2_ emissions. So I’m starting to think – or include that in my like private life as well. So now I see the sense of – I see why I can – why there’s that advantage of taking the bus, for example. Or using car sharing. Yeah, car sharing transportation instead of like – I don’t have a car myself.* (B11 more engaged group; Aarhus).

The speaker describes how working on projects to reduce carbon emissions from transportation at work, had crossed a focal boundary between work and private life, leading to them questioning their private-sphere travel-mode choices and being more aware of the merits of using more “*intelligent*” travel modes such as public transport, car-pooling schemes, as well as not owning a car. Central to the account is the idea of consistency in behavior between one context and another.

#### Recollections of Spillover Effects Between Different Cultural Contexts

Another type of contextual spillover involved exposure to wider cultural contexts beyond the workplace where pro-environmental behaviors were more socially normative than at home. With reference to contextual spillover effects from exposure to other cultures with contrasting pro-environmental behavioral norms, participants who had traveled, studied or worked overseas in countries with higher standards of environmentally responsible behavior reported a need to act consistently after returning home:

**Table:** 

Researcher:	*Was there a particular reason why you chose to start waste sorting?*
Participant [*Direct*]:	*I started in Germany. In Germany the garbage sorting is a very natural thing. So, they have a very good sorting system. When I – actually I got used to garbage sorting when I was in Germany. I feel that’s something we can do everywhere. Every citizen can participate basically. When I live in Shanghai I just feel not comfortable I mixed up things*.
Researcher:	*When you came back?*
Participant:	*If I – yeah. If we put organic waste in the same garbage bin, I don’t know, it just made me very disgusted when I saw things mixed together. I don’t know why. I just feel they should be separate…Then six years ago when we started the organic farm I realized that we have the opportunity to sort the garbage, and that we can separate – treat the compost, the organics. So I think this is one thing we can do, and that we just do it.* (B4 more engaged group; Shanghai).

Perhaps unsurprisingly, examples of this kind of contextual spillover were only found in Brazil and China, where infrastructure conducive to facilitating behaviors such as recycling was less widespread than in countries such as Denmark. This kind of example suggests that exposure to supportive pro-environmental norms and infrastructure for engagement can, at least in some cases, be internalized in ways that predispose an individual to perform that behavior in other cultural contexts, including those contexts where engagement is markedly more difficult. Processes related to behavioral consistency are central to the contextual spillover effects described in the second extract. The speaker explains how reverting to a system where waste was not recycled sparked a visceral sensation of cognitive dissonance that underpinned the spillover effect. Therefore, if behavior is internalized then it may persist in contexts where it is neither the norm, nor easy to do.

#### Recollections of Spillover Effects Between Individuals in Different Contexts

There was also very limited evidence for the second type of contextual spillover involving the transfer of behavior between different individuals across contexts. This type of spillover was reported exclusively by participants in the more environmentally engaged group in Brazil. Such accounts constructed the spillover of behaviors through social diffusion. For example, participants discussed how making changes to their homes to make them more energy-efficient had served as an exemplar for friends and neighbors, who borrowed ideas for making changes to their own homes. In addition, participants who worked in the environmental sector also spoke of how their work influenced people outside of work to become more pro-environmental as a result. In the following extract the speaker illustrates the latter kind by discussing the way in which their work potentially caused their partner to make substantial lifestyle changes without being directly influenced:

**Table:** 

Researcher:	*Do you feel like you’ve changed as a person since you started doing those (pro-environmental) behaviors?*
Participant [*Direct*]:	*Yeah, I have. For example, my partner that lives with me, he changed his lifestyle. But I don’t know if I stimulated him. I think only because I work in WWF and he start to be interested about what I was – were doing and something. I think his behavior is more sustainable than mine today.* (more engaged group; Brasília).

While the partner’s motivation to change their lifestyle is not unequivocally attributed to the speaker’s influence, an interest in the *identity* and *role* of the speaker as a WWF employee constitute the catalyst rather than behavior. The idea that the effect was not catalyzed in other ways (for example by the partner observing the speaker) is questioned in the account where the speaker suggests that it is “*only*” because of the speaker’s role and that their behavior was more sustainable than their own.

### Perceived Barriers to Behavioral Spillover

While we have focused on examples of positive behavioral spillover from the interviews, we also recognize the need to acknowledge that half of participants overall did not recall experiencing behavioral spillover. By asking participants to recall episodes of spillover verbally, there is a likelihood that some people could not recall motivation for engaging in certain actions. While little could be gleaned from responses in terms of the reasons *why* participants did not recall spillover, there were occasional utterances that offer some clues as to why behavioral spillover effects were fairly uncommon. These primarily came from interviews with less engaged participants and relate to a lack of conscious reflection on environmentally relevant practices, limited behavioral repertoires and a lack of intrinsic motivation to adopt other actions.

#### Narrow Pro-Environmental Behavioral Repertoires Inhibit Spillover Effects

There was some evidence that narrow pro-environmental behavioral repertoires may be another reason for limited spillover effects, as a scarcity of potential catalyzing actions reduces the chance of one behavior leading to another. In the following extract with a less engaged participant in Brazil, the speaker attributes their inability to recollect behavioral spillover to a lack of experience of performing pro-environmental behaviors and a lack of intrinsic motivation:

**Table:** 

Researcher:	*Can you remember a time in the past where you did one environmental behavior and it caused you to then do another environmental behavior because of the first one?*
Participant [*Transl.*]:	*He thinks that a specific behavior has not led him to do another behavior, because he hasn’t done anything in a large range. So he thinks that small things make him feel good, but it’s not like the things are leading him to do other things, because he was never stimulated to, for example, get something, a reward or something like that, because he never has done anything really big, or only specifically small actions* (A1 less engaged group; João Pessoa).

In addition to having a very narrow range of simple behaviors that, nonetheless confer a positive sense of wellbeing, the kinds of behaviors performed do not lead to others because they lack the necessary “*stimulation*” or “*reward*.” This suggests a lack of intrinsic motivation, which precludes the possibility of adopting more committed actions.

#### Lack of Reflection on Pro-environmental Behavior Inhibits Spillover Effects

When asked about whether they could recall any personal experience of spillover, participants also spoke about how they never consciously reflected on their behaviors, nor discussed them with others. Instead pro-environmental behaviors were constructed as having a routine, habitual character:

**Table:** 

Researcher:	*Can you ever remember a time in the past where you did a behavior that was good for the environment, and as a result of doing that behavior it caused you to do another behavior that was good for the environment? So one behavior leading onto another?*
Participant [*Direct*]:	*Like a chain reaction?*
Researcher:	*Yeah, yeah*
Participant:	*No, I don’t think so because it’s just habit I never actually talk about it, or think about, it’s just things that I do…* (A9 less engaged group; Aarhus).

Whereas accounts of behavioral spillover tended to highlight the salience of conscious awareness of behavior (including environmental impacts, alignment with broader goals and consistency with other behaviors), accounts such as the above that attempt to account for a lack of recollection of spillover provide a counterpoint. In contrast, they describe how spillover may have been impeded by a lack of conscious reflection on the perfunctory action being performed, particularly in terms of that action’s relationship to other behaviors. This is also suggested in terms of the character of the behavior itself, in which pro-environmental actions are “*just things that I* do” as opposed to practices with the intention of reducing one’s environmental impact.

## Discussion

This paper offers an original qualitative analysis of subjective accounts of behavioral spillover in three diverse cultural contexts. Our research questions set out to address 5 research questions; whether citizens in different countries reported experiencing behavioral spillover; whether there were any differences in reports of spillover between different cultures; whether there were any differences based on level of environmental engagement; what kinds of spillover effects were reported; and whether any potential barriers to spillover existed.

### Evidence for Positive Behavioral Spillover in Personal Accounts Across Cultures

Reflecting previous (mainly quantitative) work on behavioral spillover (Truelove et al., 2014; Nash et al., 2017), behavioral spillover effects were found in all three cultural contexts. In line with our first research question, overall, our analysis showed that half of participants who were directly questioned recalled an experience they considered analogous to positive behavioral spillover. However, these accounts did not arise spontaneously in interviews but were elicited through direct questioning. Furthermore, not all accounts of behavioral spillover could be defined as such, as a proportion did not involve one behavior being catalyzed by another behavior. Instead, alternative behavioral motivations that did not match conventional definitions of spillover (such as formative experience and significant life changing events) came up in some responses, reflecting lay conceptions that are less clearly defined and do not map precisely onto conventional scholarly schematics (Rudiak-Gould, 2012).

### Differences in Personal Accounts of Positive Behavioral Spillover Between Cultures

Across the three cultures we found relatively few clear differences in accounts of spillover experience, which may at least partly reflect the relative infrequency of clear and detailed accounts of behavioral spillover and a methodological approach that relied on participant recall. In addition, this may also be a function of the rather narrow pro-environmental behavioral repertoires practiced by many participants. Perhaps surprisingly, participants in Denmark were less likely to recall spillover than those in China and Brazil. However, given the relatively low frequencies of spillover effects, further investigation with larger sample sizes would be useful to draw out cultural differences.

In all three countries, within-domain spillovers were the most commonly reported effects, involving the transfer of household practices within the same behavioral cluster (mainly limited to clusters involving waste or resource conservation), or an increase in the frequency or range of existing actions. In addition to catalyzing similar behaviors, wider engagement on sustainability issues with other people was also catalyzed.

While between-domain, contextual and interpersonal spillover effects were also reported, their relative infrequency made it difficult to judge whether cultural differences existed; though there were indications that contextual spillovers were more common in China, while interpersonal spillover effects were only reported in Brazil. This could reflect cultural differences in terms of construal. Work on cultural values (Markus and Kitayama, 1991) asserts that differences in cultural self-construal affect the way in which individuals understand the self in relation to others. While individuals in North American and Northern European cultures see the self as more independent from others, in Asian and African cultures the self is more interdependent with others. Studies have found interdependent self-construal to be predictive of greater ecological cooperation than independent self-construal (Arnocky et al., 2007). Therefore, in promoting forms of spillover involving social diffusion, it may be necessary to take cultural barriers into consideration. Additional work with larger sample sizes is needed to elaborate on these potential cultural differences and address existing gaps.

In line with research question 2, such indications suggest, but do not in themselves confirm the presence of cultural differences. With reference to our methodological approach, there is also the potential that the phrasing of the question designed to elicit spillover was unclear and potentially culturally biased, generating a narrow range of responses (Shiraev and Levy, 2016). A more culturally sensitive approach might have done more to tailor questions more sensitively to each cultural context, though this would have made comparability more problematic. Further exploration and more careful follow-up questioning might have also uncovered more culturally specific nuance.

### Differences in Personal Accounts of Positive Behavioral Spillover and Environmental Engagement

The clearest differences in reports of spillover were linked to environmental engagement rather than culture. Following research question 3, participants who were more environmentally engaged were far more likely to recall spillover regardless of country. This was also the case regardless of the type of spillover reported. Based on consistent observed differences, pre-existing pro-environmental values appear to facilitate spillover (see also Thøgersen and Crompton, 2009). Those who prioritize the environment to some degree appeared to reflect upon behaviors with a more environmental focus, in contrast to those who were less engaged and viewed the things they did as simply part of the everyday routine. It may be that more environmentally engaged citizens are more consciously aware of the impacts of the behaviors they perform (Kollmuss and Agyeman, 2002), more consistent in their behavior in line with perceived self-identity (Cialdini et al., 1995), or more driven by concern to do something to address environmental problems (Steg and Vlek, 2009).

### Differences in Reported Positive Behavioral Spillover Effects

Within the interviews across cultures an array of positive spillover effects were reported. We now reflect on the nature of these effects separately.

#### Within-Domain Behavioral Spillover Effects

The relative frequency of within-domain spillover supports previous work proposing that behavioral spillover is likelier when behaviors are similar (Thøgersen, 2004), or share the same routines or resources (Littleford et al., 2014; Margetts and Kashima, 2017). Within-domain spillovers may also require less effort. This parallels other research measuring a gradual expansion of organic food purchasing using supermarket loyalty card and scanner data (as opposed to less robust self-report measures) (Juhl et al., 2017).

While there was some commonality of behavioral clusters leading to reported spillover effects, there was little clarity as to which specific behaviors catalyzed others. It appears unlikely that specific behaviors function as entry points to adopting other actions. There was also little evidence that easier behaviors lead to more committed ones. While unsupported by our analysis, this may be due to a lack of self-efficacy. Increased self-efficacy has been demonstrated not only as a motivator of environmentally responsible action, but as a mediator of further engagement in wider behaviors (Lauren et al., 2018) and warrants further study. There was also some evidence that engaging in one behavior catalyzed wider interpersonal engagement. This might be more effective in generating wider culture change than focusing on spillovers involving the adoption of individual behaviors. This also parallels other work in which it is argued that engagement in green behavior catalyzing pro-environmental policy support has greater potential impact than conventional spillovers between behaviors (Thøgersen and Noblet, 2012).

#### Between-Domain Behavioral Spillover Effects

Behavioral interventions potentially risk marginal returns if behavioral repertoires are limited to “simple and painless” actions (Thøgersen and Crompton, 2009). It is also evident from everyday life that if we engage in one environmentally responsible action then this does not guarantee that we will then engage in other behaviors, akin to ascending a “*virtuous escalator*” (Thøgersen and Crompton, 2009). We found very little evidence across cultures where a behavior from one cluster catalyzed another behavior from a different cluster. If spillover is to fulfill its potential in generating wider lifestyle change, interventions must find ways to transcend these boundaries and catalyze the voluntary adoption of wider, more committed practices beyond existing behavioral repertoires.

From our isolated examples of between-domain spillover, an initial step could lie in highlighting the co-benefits of pro-environmental engagement (such as promoting health or voluntary simplicity). This is not to say that environmental justifications for engagement are less important, as without some degree of intrinsic pro-environmental motivation, spillover may be undermined if a perceived benefit or incentive disappears (Evans et al., 2013). Nonetheless, some individuals will value the environment more than others and so strengthening pro-environmental salience in decision-making across the board is likely to be extremely difficult. Research has shown that certain types of co-benefits (for example, the creation of more benevolent and caring communities) can motivate sustainable behavior change for those who are environmentally committed to varying degrees (Bain et al., 2016). This could create the initial momentum for change. Further to the above, catalyzing wider interpersonal engagement might also be an effective way of generating wider culture change.

#### Contextual Behavioral Spillover Effects

We also found evidence for contextual spillover effects. Previous studies have also documented the transfer of behavior between different contexts including work and home (Lee et al., 1995; Tudor et al., 2007; Andersson et al., 2012). This opens the possibility that promoting pro-environmental practices at work could be spread to other life spheres. However, as the examples from the interviews came from participants who worked in the environmental sector, it is possible that pre-existing values could have facilitated consistency (Thøgersen, 2012). Other work has found contextual spillover mediated via identification with the pro-environmental ethos or values within non-environmental organizations (Rashid and Mohammad, 2011; Loverock et al., 2015). Workplace coercion might also lead to behavioral transfer from the workplace to the home, which could influence less environmentally engaged employees. Andersson et al. (2012) report increases in home waste separation practices following the introduction of an environmental management system at work.

Interpersonal spillover effects were also reported that operated along processes of *social diffusion* (McKenzie-Mohr and Schultz, 2014). Taken together, the evidence suggests that if behavior can be transferred between contexts then it might also then be spread via social diffusion to multiple people within the household under the right conditions. There is also the possibility of a reversal of direction from home to work, though differences in roles, levels of responsibility and control in the workplace might constrain the degree to which household practices could transfer to the workplace (Maki et al., 2016).

Following the lead of CBSM, rather than attempting to identify and promote the adoption of what are judged to be the most potent behavioral catalysts, it may be more productive to tailor interventions based on the receptiveness of different audiences. It may be that in some situations different kinds of spillover pathways will be open or closed. A better understanding of the ways in which different types of spillover operate would be a suitable target for CBSM interventions. In particular, CBSM strategies could utilize community connections and block leaders to create small-scale cultural shifts that can grow and spread through society (McKenzie-Mohr et al., 2011). Small-scale community approaches might also be more successful in reaching those who feel that they lack the capacity to engage in more committed pro-environmental actions, as indicated in the interviews. Community initiatives that foster supportive environments in which more sustainable behaviors can develop, may also be more impactful than individual private-sphere initiatives that ignore the relevance of the social context.

### Barriers to Positive Behavioral Spillover

Conscious awareness and personal importance of the initial behavior catalyzing spillover was significant in multiple accounts of spillover, which came from more engaged participants. Much of our day-to-day behavior is not consciously performed (Carden and Wood, 2018), which suggests that behavioral spillover may be impeded by a lack of conscious attention to routinized behavioral decisions, especially for those who were less engaged. Environmental considerations may also be subjugated by more pressing day-to-day concerns and responsibilities that characterize the life of the average citizen. Behaviors like recycling can blend into everyday routines over time, losing their environmental significance (Cornelissen et al., 2008; Thomas and Sharp, 2013), thereby reducing the possibility of spillover. Generating greater conscious awareness of the environmental significance of behavior could therefore strengthen an action’s catalyzing potential. Barbaro and Pickett (2016) report positive associations between mindfulness, sense of connectedness to nature, and engagement in a range of pro-environmental behaviors. In line with the habit discontinuity hypothesis (Verplanken et al., 2008), interrupting behavioral routines can reinvigorate awareness and promote more sustainable behavioral choices.

### Study Limitations and Future Research

Like all other studies, there are limitations of the methods applied here. The use of a single qualitative method alone can provide only a partial picture of spillover, which rests on subjective self-report and not actual behavior over time. Recollection of motivations *after* behavior has taken place may be subject to distortion and post-rationalization as individuals try to piece together their motives, especially if behavior occurred sometime in the past (Broemer et al., 2008). Clearly establishing causal links between behaviors is especially problematic due to the many factors governing decision-making, not all of which an individual will be conscious of. The reasons for maintaining a behavior may also be different from the reasons for beginning a behavior.

The rather limited evidence for behavioral spillover found not only in this study but across much of the literature brings into question how to proceed in future research into spillover. The core assumption of spillover as a means of initiating voluntary and cumulative behavior change has given way to a more complex and contingent perspective, in which spillover takes multiple forms, in which certain behaviors may be catalyzed for certain individuals in certain contexts. As previously discussed, interventions that target spillover processes aiming to catalyze wider social engagement may offer greater potential than interventions targeting narrower changes to individual practices. Mixed method approaches should also be employed to measure behavioral outcomes utilizing rigorous quantitative methods longitudinally and capturing the richness and detail of more qualitative techniques (Verfuerth and Gregory-Smith, 2018). We also encourage the application of qualitative approaches (for example, focus group discussions) with more and less environmentally engaged groups to identify obstacles and facilitators of behavior change for different groups across cultural contexts, including spillover processes. Approaches involving groups could incorporate a wider repertoire of CBSM steps and tools tailored to the individual characteristics of those groups. Future research might also examine reflections on behavior change processes *as they occur*, rather than *after* they have occurred. In addition, to shedding light on factors that create conditions favorable to spillover, greater attention to cases where behavioral engagement does not lead to other actions might also uncover processes hitherto concealed from the attention of social scientists.

## Ethics Statement

This study was carried out in accordance with the recommendations of “name of guidelines, name of committee” with written informed consent from all subjects. All subjects gave written informed consent in accordance with the Declaration of Helsinki. The protocol was approved by the Ethics Committee, School of Psychology, Cardiff University.

## Author Contributions

All authors listed have made a substantial, direct and intellectual contribution to the work, and approved it for publication.

## Conflict of Interest Statement

The authors declare that the research was conducted in the absence of any commercial or financial relationships that could be construed as a potential conflict of interest.

## Footnotes

1 Response options for gender comprised “Female,” “Male” and “Other/Prefer not to say.”
